# Uptake and Survival of African Swine Fever Virus in Mealworm (*Tenebrio molitor*) and Black Soldier Fly (*Hermetia illucens)* Larvae

**DOI:** 10.3390/pathogens12010047

**Published:** 2022-12-28

**Authors:** Ann Sofie Olesen, Christina Marie Lazov, Antoine Lecocq, Francesc Accensi, Annette Bruun Jensen, Louise Lohse, Thomas Bruun Rasmussen, Graham J. Belsham, Anette Bøtner

**Affiliations:** 1 Department of Virus & Microbiological Special Diagnostics, Statens Serum Institut, DK-2300 Copenhagen, Denmark; 2 Department of Veterinary and Animal Sciences, University of Copenhagen, DK-1870 Frederiksberg, Denmark; 3 Department of Plant and Environmental Sciences, University of Copenhagen, DK-1870 Frederiksberg, Denmark; 4 Unitat Mixta d’Investigació IRTA-UAB en Sanitat Animal, Centre de Recerca en Sanitat Animal (CReSA), Campus de la Universitat Autònoma de Barcelona (UAB), 08193 Bellaterra, Spain; 5 Departament de Sanitat i d’Anatomia Animals, Facultat de Veterinària, Campus de la Universitat Autònoma de Barcelona (UAB), 08193 Bellaterra, Spain

**Keywords:** African swine fever virus, black soldier fly, feed safety, insect rearing, mealworm, virus survival, virus transmission

## Abstract

Insect production offers a sustainable source of nutrients for livestock. This comes with a risk for transmission of pathogens from the insects into the livestock sector, including viruses causing serious diseases, such as African swine fever virus (ASFV), classical swine fever virus and foot-and-mouth disease virus. ASFV is known to survive for a long time within animal meat and byproducts. Therefore, we conducted experimental exposure studies of insects to ASFV using larvae of two key insect species produced for food and feed, the mealworm; *Tenebrio molitor*, and the black soldier fly, *Hermetia illucens*. The larvae were exposed to ASFV POL/2015/Podlaskie, via oral uptake of serum or spleen material from ASFV-infected pigs. Using qPCR, the amounts of viral DNA present immediately after exposure varied from ~10^4.7^ to 10^7.2^ genome copies per insect. ASFV DNA was detectable in the larvae of *H. illucens* for up to 3 days post exposure and in *T. molitor* larvae for up to 9 days post exposure. To assess the presence of infectious virus within the larvae and with this, the risk of virus transmission via oral consumption, pigs were fed cakes containing larvae exposed to ASFV. Pigs that consumed 50 *T. molitor* or 50 *H. illucens* virus-exposed larvae did not become infected with ASFV. Thus, it appears, that in our experimental setting, the risk of ASFV transmission via consumption of unprocessed insect larvae, used as feed, is low.

## 1. Introduction

Insect production offers a sustainable source of nutrients [1] as insect larvae can efficiently convert a broad range of biomass to valuable proteins (including essential amino acids), fats, vitamins and minerals. According to the *International Platform of Insects for Food and Feed (IPIFF),* by 2025, most of the demand for insect meal will lie in the pet food sector (approximately 40–50% of the insect meal produced). Subsequently, the growth in use of insect derived processed animal proteins (PAPs) as aquaculture feed will continue reaching 25–35% in terms of market share. Similarly, the market share for insects as feed for poultry (to 20–30%) and for pigs (to 5–15%) are also expected to increase rapidly [2], following the end of the ban (in the EU) on using PAPs from insects in these sectors [3]. However, still, only a limited range of substrates can be used for rearing of the insects used for feed and food [4], and using waste materials that contain animal byproducts as feed for the insects is not allowed [4,5]. To increase the sustainable circular economy potential of insect production, novel, currently banned [4] sources of substrates for insect rearing should be explored. Such substrates include food waste containing meat, slaughter byproducts and animal manure [1]. In order to assess the safety of insects as food and feed when using such substrates of animal origin, knowledge regarding the fate of different, potentially hazardous, agents from animal products in the insects is required. Such agents could be transmitted to animals and humans when they consume the insects or insect-derived products. These hazards can be of chemical (toxins, heavy metals, pesticides) or microbiological (bacteria, prions, fungi, parasites, viruses) origin [5]. For viruses, special concern can be raised for potential transmission of the agents responsible for serious notifiable diseases that are known to be transmitted efficiently via animal products and to survive within meat for a prolonged time period, including African swine fever virus [6], classical swine fever virus [7] and foot-and-mouth disease virus [8,9]. Currently, African swine fever virus (ASFV), in particular, is of major global concern as this virus has now reached pandemic proportions, affecting a number of countries within eastern and western Europe, Asia, the Caribbean and Sub-Saharan Africa [10]. Hence, even within Europe, there is a risk for this virus to be present within different animal byproducts that could be used as a substrate for insect larvae production if the EU legislation is changed. 

Experimental exposure studies of insects used for feed and food with animal pathogens, including viruses, can provide knowledge about the survival of these pathogens within production systems. Such knowledge can be used for future risk assessments of insect rearing on substrates containing products of animal origin. Previously, we used virus bioexposure assays to examine the uptake and survival of viruses in larvae of two of the most widely used insect species for the production of food and feed, the mealworm (*Tenebrio molitor*) and the black soldier fly (*Hermetia illucens*) [5,11], using porcine respiratory coronavirus as a model [12]. In the current study, similar assays were applied to test the survival of ASFV in larvae of these two insect species. Insects containing ASFV, following oral uptake, were subsequently fed to pigs in order to investigate potential transmission of the virus to susceptible host animals via these larvae. 

## 2. Materials and Methods

### 2.1. Insect Exposures 

#### 2.1.1. Exposure Viruses 

For experimental exposure of the insect larvae and for positive controls, samples obtained from pigs infected with ASFV POL/2015/Podlaskie were used [13,14]. Serum was used for *T. molitor* exposures, while clarified spleen suspension (10% *w*/*v*) was used for *H. illucens* exposures. Briefly, clarified spleen suspensions were prepared in Minimum Essential Medium (MEM, Gibco, Thermo Fisher Scientific, Waltham, MA, USA) supplemented with streptomycin (Sigma-Aldrich, St. Louis, MO, USA), neomycin (Sigma-Aldrich), amphotericin (Sigma-Aldrich) and benzylpenicillin (Sigma-Aldrich). Pig spleen samples (ca. 0.1 g) were homogenized using two 3-mm stainless steel beads (Dejay Distribution Ltd., Launceston, UK) in a TissueLyser II (QIAGEN, Hilden, Germany) for 2 min at 25 Hz. The homogenates were centrifuged and supernatants collected. 

The titer of the serum pool and clarified spleen suspension used for inoculation were determined by end-point titration in porcine pulmonary alveolar macrophages (PPAM) seeded into 96-well plates (Thermo Fisher Scientific, Waltham, MA, USA). These cells were prepared as previously described [13,15]. After three days of incubation, at 37 °C in an atmosphere with 5% CO_2_, virus infected cells were identified following fixation and staining of the cells using an immunoperoxidase monolayer assay (IPMA) [13,15]. Briefly, the cells were stained using ASFV antibody positive swine serum and protein A-conjugated horseradish peroxidase (Sigma-Aldrich), together with hydrogen peroxide and 3-amino-9-ethyl carbazole (Sigma Aldrich) as chromogenic substrate. Red-stained cells were counted using a light microscope and virus titers were calculated using the method described by [16]. 

#### 2.1.2. Experimental Insects

The *T. molitor* larvae were sourced from a population reared at the Section for Organismal Biology (SOBI) facility of the University of Copenhagen (UCPH), Denmark. The larvae were housed in plastic containers (16.5 × 10 × 7 cm) fitted with a vented lid. They were reared at 27 °C and 60–70% relative humidity in darkness. For weight and size consistency, all larvae used for the virus exposure bioassays were 8 ± 1 weeks old and weighed on average 100 ± 20 mg per individual. The larvae were reared on ground oats provided ad libitum and cut potatoes, provided as a water source, were replaced every 3 days.

The *H. illucens* larvae were supplied by a commercial producer, ENORM, Denmark. The larvae were housed in plastic containers (16.5 × 10 × 7 cm) fitted with a lid containing a mesh covered surface to avoid escapes. For weight and size consistency, 6-day-old larvae were used for the virus exposure. The larvae were reared on wet chicken feed (GOLD 4 GALLICO, Versele-Laga pellets in tap water in a ~1:1 ratio (*w*/*w*)). 

#### 2.1.3. Feeding of Insects

Feeding of insects with ASFV was performed within high containment facilities at the Statens Serum Institut (SSI, Copenhagen, Denmark). The insect larvae were kept for a minimum of one day at the research facility prior to virus exposure for acclimatization. 

*Tenebrio molitor* larvae were allowed to feed on 5 µL cell culture medium (non-exposed larvae) or 5 µL serum from a pool of serum obtained from pigs infected with ASFV POL/2015/Podlaskie (ASFV-exposed larvae) in two separate studies (studies T1 and T2). Exposures were performed as previously described for *T. molitor* virus exposures using porcine respiratory coronavirus [12]. Briefly, following 24 h solitary housing without access to food or water in plastic medicine cups, the larvae were exposed to the medium or serum. Following ~5–15 min exposure and visual examination to assess uptake of the liquid and any outside contamination of the larvae [12], larvae that had consumed the liquid without visible contamination were moved to two plastic containers (as described above), one for non-exposed larvae and one for larvae exposed to ASFV. The larvae were housed in an environmental chamber at 27 °C and 60–70% relative humidity. A Falcon tube containing an aliquot of the same serum pool used for exposures of the larvae was incubated under the same conditions. 

*Hermetia illucens* larvae were exposed to feed spiked with cell culture medium (non-exposed larvae) or feed spiked with clarified spleen homogenate obtained from pigs infected with ASFV POL/2015/ Podlaskie (ASFV-exposed larvae) in two studies (studies H1 and H2). Exposures were performed as previously described for *H. illucens* virus exposures [12]. For ASFV exposures, 20 g wet chicken feed spiked with clarified spleen suspensions (10% *w*/*v*) was used. Briefly, chicken feed pellets were mixed thoroughly with the clarified spleen suspension, and water was added to obtain the desired texture. After the addition of water, once again the final feed was thoroughly mixed. Following 2 h or 24 h (study H1) and 5 h or 24 h (study H2) exposure to the spiked feed, the larvae were moved to fresh (un-spiked) feed. The larvae were housed in two plastic containers, as described above, one for control (non-exposed) larvae and another for the virus exposed larvae. The larvae were housed in an environmental chamber at 27 °C and 60–70% relative humidity. A Falcon tube containing the same clarified spleen suspension used for insect exposures and the spiked chicken feed (without larvae) was incubated under the same conditions. 

#### 2.1.4. Sampling and Euthanasia

For sampling of *T. molitor*, 10 unexposed larvae, 25 exposed larvae and an aliquot of the serum pool were transferred individually into 2 mL Eppendorf tubes at set time points following exposure. In study T1, samples were collected at 0 days post exposure (dpe), up to 30 min after virus ingestion, and subsequently at 1, 2, 3, 4, 6 and 9 dpe. In study T2, samples were collected at 0, 1, 2, 3, 4 and 7 dpe. 

For sampling of *H. illucens*, 10 unexposed larvae, 25 exposed larvae, a sample of spiked feed and an aliquot of the clarified spleen suspension were transferred individually into 2 mL Eppendorf tubes at set time points following exposure. In study H1, samples were collected at 2 h and 24 h into exposure to the spiked feed, and subsequently at 1, 2, 3 and 5 dpe following removal of the larvae from the spiked feed. In study H2, samples were collected at 5 h and 24 h into exposure to the spiked feed, and then at 1, 2, 3 and 6 dpe following removal of the larvae from the spiked feed. In studies H1 and H2, spiked feed that the *H. illucens* larvae had been housed in was sampled at 24 h, in addition to spiked feed samples from the incubator (that had not been exposed to the larvae). On the other time points, only spiked feed that had not been exposed to the larvae was sampled. Following collection, the larvae were euthanized by freezing (−80 °C). The larvae, the collected spiked feed, serum and clarified spleen suspensions were stored at −80 °C until further processing and analyses. 

In addition to the serum pools and clarified spleen suspension obtained from the insect incubator (27 °C, 60–70% RH) as described above, 1.5 mL Eppendorf tubes containing 0.5 mL of serum or clarified spleen suspensions were kept in a Thermomixer (Eppendorf) at 27 °C. The tubes were collected after 0 h, 2 h, 5 h, 24 h, 48 h, 72 h and 96 h storage at 27 °C in two subsequent studies for both serum and spleen samples. The samples were stored at −80 °C until further processing and analyses. 

#### 2.1.5. Pre-Processing of Samples 

After thawing, *H. illucens* larvae were rinsed externally to collect wash fluids. Minimum Essential Medium (MEM, Gibco) (1 mL) supplemented with streptomycin (Sigma-Aldrich), neomycin (Sigma-Aldrich), amphotericin (Sigma-Aldrich) and benzylpenicillin (Sigma-Aldrich) was added to each sample of *T. molitor* larvae, rinsed *H. illucens* larvae and spiked feed. The samples were homogenized using two 3-mm stainless steel beads (Dejay Distribution Ltd.) in a TissueLyser II (QIAGEN) for 2 min at 25 Hz. The homogenates were centrifuged and supernatants were collected for further analyses. 

#### 2.1.6. ASFV DNA Detection by Quantitative Real-Time Polymerase Chain Reaction (qPCR)

Nucleic acids were purified from larvae homogenate supernatants from both larvae species (diluted 1:5 in MEM), *H. illucens* wash fluids (diluted 1:5 in MEM), spiked feed homogenate supernatants (diluted 1:5 in MEM), the collected serum pools (undiluted) and the clarified spleen suspensions (undiluted) using the MagNA Pure 96 system (Roche, Basel, Switzerland) [13]. The extracted samples were analyzed for the presence of ASFV DNA by qPCR using the CFX Opus Real-Time PCR System (Bio-Rad, Hercules, CA, USA), essentially as described by [17]. A positive result in the qPCR was defined as a threshold cycle value (Cq) at which FAM dye emission appeared above background within 42 cycles. Results are presented as viral genome copy numbers/per mL calculated by reference to a standard curve based on a 10-fold dilution series of a pVP72 plasmid [18]. 

#### 2.1.7. Virus Detection by Virus Isolation

The titers of infectious ASFV in serum and clarified spleen suspensions kept in the insect incubator at 27 °C and in the Thermomixer in the laboratory 27 °C were determined by end-point titration in PPAM as described above. 

The presence of infectious virus in six selected *T. molitor* homogenate samples from study T2 containing ASFV DNA (with Cq values from 29.8 to 32.8) was determined using PPAM. The cells were maintained in MEM with 5% fetal bovine serum (FBS) and seeded in 24 well NUNC 24-well plates (Thermo Fisher Scientific). Larval homogenate (100 µL) was mixed with 100 µL MEM with 10% FBS, streptomycin (Sigma-Aldrich), neomycin (Sigma-Aldrich), amphotericin (Sigma-Aldrich) and benzylpenicillin (Sigma-Aldrich) and inoculated onto 1 mL cells (1,600,000 cells/mL). The inoculum was removed after one hour incubation at 37 °C (in 5% CO_2_) and the cells were washed twice using 1x PBS. Following washing, 1 mL MEM containing 5% FBS, streptomycin, neomycin, amphotericin (Sigma-Aldrich) and benzylpenicillin was added to the cells. 

After three days of incubation at 37 °C (in 5% CO_2_), the cells were harvested by freezing and 100 µL of the 1st passage material was inoculated onto 1 mL fresh PPAM. After three days of incubation, virus-infected cells were identified using the IPMA as described above (Section 2.1.1). 

### 2.2. Pig Exposures 

#### 2.2.1. Pigs

The experiment in pigs was performed within high containment facilities at the Centre de Recerca en Sanitat Animal (IRTA-CReSA, Barcelona, Spain).

Twenty-four pigs (12 males and 12 females), eight weeks of age, were included in this study. The pigs were obtained from a conventional Spanish swine herd (Landrace X Large White). On arrival at the research facility, one week before the start of the experiment, all pigs were found to be healthy by veterinary inspection. Water and a commercial diet for weaned pigs were provided ad libitum.

Animal care and maintenance, experimental procedures and euthanasia were conducted in accordance with EU legislation on animal experimentation (EU Directive 2010/63/EU). The study was approved by the Ethical and Animal Welfare Committee of the Generalitat de Catalunya (Autonomous Government of Catalonia; permit number: CEA-OH/11744/2).

#### 2.2.2. Challenge Material 

*Tenebrio molitor* larvae and *H. illucens* larvae fed as described in Section 2.1.3. were used for inoculation of pigs. *Tenebrio molitor* larvae used for virus exposure were euthanized by freezing at −80 °C immediately after virus exposure and at 48 h after exposure, while *H. illucens* larvae were euthanized in the same manner following 5 h or 24 h exposure to virus-spiked feed. Unrinsed larvae were added to ~100 g soft cake that was then eaten by each pig (as described for *Stomoxys calcitrans* [14]).

#### 2.2.3. Study Design

Upon arrival at the research facility, the pigs were allocated into four pens, each containing six pigs, within two containment stable units (BSL-3). After an acclimatization period of one week and ~5 h fast, the pigs were individually fed the cakes containing the larvae. 

In group 1, six pigs (pigs 1–6) were individually fed soft cakes containing 50 *T. molitor* larvae that had been euthanized immediately after feeding on serum containing ASFV. In group 2, six pigs (pigs 7–12) were fed cakes containing 50 *T. molitor* larvae that had been euthanized 48 h after feeding on this serum. Group 3 (pigs 13–18) were fed cakes containing 50 *H. illucens* larvae that had been euthanized after 5 h feeding on feed spiked with clarified spleen suspension containing ASFV. Group 4 (pigs 19–24) were fed cakes containing 50 *H. illucens* larvae that had been euthanized 24 h after feeding on the same spiked feed. 

#### 2.2.4. Clinical Examination and Euthanasia 

Clinical scores and rectal temperatures were recorded from individual pigs on each day (up to 13 days post feeding). A total clinical score was calculated per day based on a previously described system [18]. The pigs were euthanized at the end of the study period by intravascular injection of Pentobarbital following deep anesthesia.

#### 2.2.5. Pig Sampling

EDTA-stabilized blood (EDTA blood) and unstabilized blood samples (for serum) were collected prior to oral exposure at 0 dpe and at 13 dpe. 

#### 2.2.6. ASFV DNA Detection by Quantitative Real-Time Polymerase Chain Reaction (qPCR)

EDTA blood was tested for the presence of ASFV DNA as described in Section 2.1.6. 

#### 2.2.7. Antibody Detection 

Sera obtained at euthanasia were tested for the presence of anti-ASFV antibodies using an Ingezim PPA Compac ELISA (^®^INGENASAINGEZIMPPA COMPAC K3 INGENASA) according to the manufacturer’s instructions.

### 2.3. Descriptive Statistics

For genome copy numbers obtained in samples immediately following exposure in the four studies (at 0 dpe), median (M), upper (Q1) and lower (Q3) quartiles were calculated using the QUARTILE function in Excel (2016). The values are reported as (Q1, M, Q3). The values were calculated after the conversion of log_10_ genome copy numbers to the direct scale through exponentiation. 

## 3. Results

Titration of the inoculum established that each *T. molitor* larva was exposed to 10^3.3^ TCID_50_ ASFV in 5 μL serum. Titration results obtained for clarified spleen suspension used for feed inoculation were applied to calculate the theoretical titer of the spiked feed used for exposure of *H. illucens* to be ~10^5^ TCID_50_ ASFV/g feed. During all four studies, no obvious symptoms (lethargy, discoloration, behavioral changes) and no mortality were observed among the *T. molitor* and *H. illucens* larvae following exposure to ASFV. 

### 3.1. Detection of ASFV in T. molitor Larvae and Serum 

**Figure 1:**
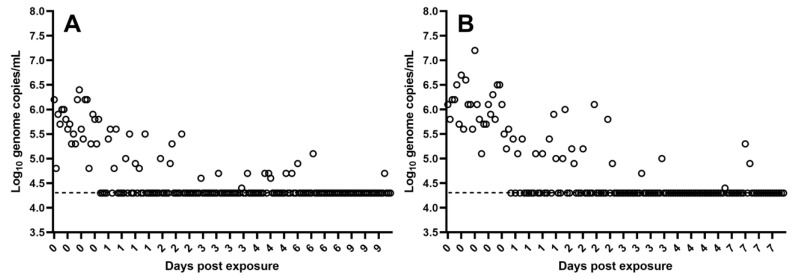
The presence of ASFV DNA in the *T. molitor* larvae, in study T1 at 0 days post exposure (dpe) and subsequently at 1, 2, 3, 4, 6 and 9 dpe (**A**) and in study T2 at 0, 1, 2, 3, 4 and 7 dpe (**B**), was quantified by qPCR and values converted to log_10_ genome copy numbers/larvae homogenate (mL) using a standard curve. Levels below 10^4.3^ ASFV genomes/mL were below the detection limit (indicated by dashed line) of the assay.

Using qPCR, ASFV DNA was detected in 96–100% of the *T. molitor* larvae sampled after exposure at 0 dpe in studies T1 and T2 (Table 1). In these larvae, ASFV genome copies/1 mL varied between 10^4.8^ and 10^6.4^ (Q1 = 10^5.4^, M = 10^5.7^, Q3 = 10^6.0^, n = 24) (in study T1, Figure 1A) and between 10^5.1^ and 10^7.2^ (Q1 = 10^5.8^, M = 10^6.1^, Q3 = 10^6.3^, n = 25) (in study T2, Figure 1B). Using virus isolation in PPAM, no infectious ASFV was detected in the six *T. molitor* larvae sampled at 0 dpe in study T2 that had been shown to contain ASFV DNA. In the majority of these samples, the presence or absence of infectious virus could not be determined due to the presence of contaminators (fungi, bacteria). Filtration of the sample homogenates prior to inoculation of the cells using 0.45 µM syringe filters was attempted, but proved difficult, i.e., the filters clogged.

In study T1, ASFV DNA was detected in 36% of the larvae sampled at 1 dpe, and in 4–24% of the larvae obtained from 2–9 dpe (Table 1). From 1–9 dpe, ASFV DNA levels ranged from 10^4.4^ to 10^5.6^ ASFV genome copies/1 mL in the larvae in which ASFV DNA could be detected (Figure 1A). 

In study T2, ASFV DNA was detected in 44% of the larvae sampled at 1 dpe, and in 4–32% of the larvae sampled from 2–7 dpe (Table 1). From 1–7 dpe, the levels of ASFV DNA ranged from 10^4.4^ to 10^6.1^ ASFV genome copies/1 mL in the larvae in which ASFV DNA could be detected (Figure 1B).

No viral DNA was detected in the control larvae (larvae allowed to consume cell culture medium) as expected (data not shown). 

**Figure 2:**
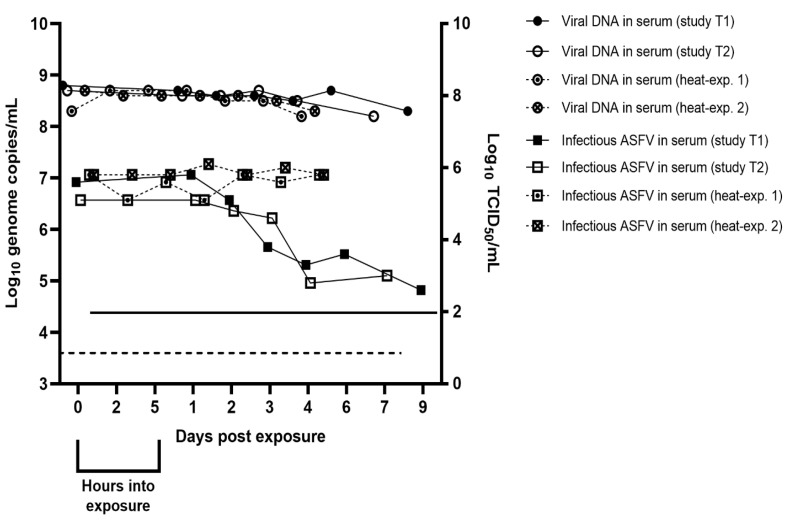
The presence of ASFV DNA and infectious virus in sampled serum controls. The dashed line indicates the detection limit for the qPCR assay (log_10_ 3.6 copies/mL). The solid line indicates the detection limit for the virus isolation assay (log_10_ 2 TCID_50_/mL).

The level of viral DNA in the serum kept in the environmental chamber from 0–6 dpe (study T1) and 0–7 dpe (study T2) at 27 °C was stable with no apparent decrease throughout the two studies. ASFV genome copies/mL in the serum samples were between 10^8.3^ to 10^8.8^ (in study T1) and 10^8.2^ to 10^8.7^ (in study T2) (Figure 2). These serum samples had an infectivity titer of log_10_ 5.6 TCID_50_/mL (study T1) and log_10_ 5.1 TCID_50_/mL (study T2) at 0 dpe. The level of infectious virus remained relatively stable, with only a very small decrease in virus titer until 2–3 dpe (study T1) or 3–4 dpe (study T2) when the levels of infectious virus dropped by 1.3 (study T1) and 1.8 (study T2) log_10_ TCID_50_/mL between samplings (Figure 2). 

The amount of ASFV DNA in the serum samples kept in the Thermomixer (Eppendorf) from 0–4 dpe (heat experiments 1 and 2) at 27 °C was stable with no apparent decrease throughout the two experiments. Genome copies/mL in the sampled serum were between 10^8.2^ to 10^8.7^ (heat-exp. 1) and 10^8.3^ to 10^8.7^ (heat-exp. 2) (Figure 2). The serum sample had a titer of log_10_ 5.8 TCID_50_/mL (heat-exp. 1 and heat-exp. 2) at 0 dpe and the level of infectious virus remained stable throughout the study period in both experiments (Figure 2). 

### 3.2. Detection of ASFV in H. illucens Larvae, Spiked Feed Samples and Spleen Suspensions 

**Figure 3:**
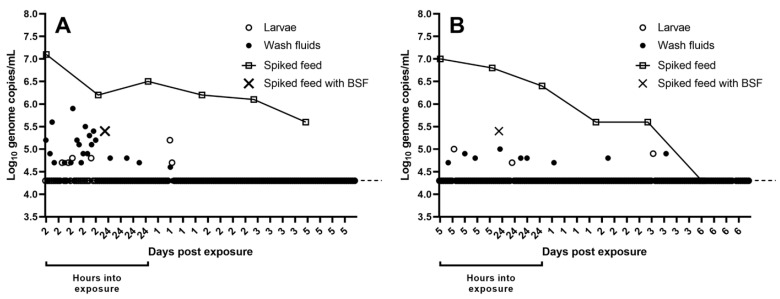
The presence of ASFV DNA in the rinsed *H. illucens* larvae, wash fluids and spiked feed samples in study H1 (**A**) after 2 h and 24 h exposure and at 1, 2, 3 and 5 dpe and in study H2 (**B**) after 5 h and 24 h exposure and at 1, 2, 3 and 6 dpe was quantified by qPCR and values converted to log_10_ genome copy numbers/sample (mL) by reference to a standard curve. Levels below 10^4.3^ ASFV genomes/mL were below the detection limit (indicated by dashed line) of the assay. BSF = black soldier fly.

Using qPCR, ASFV DNA was detected in 16% of the rinsed larvae and 68% of the wash fluids collected in study H1, following 2 h exposure to feed spiked to a titer of ~10^5^ TCID_50_ ASFV/g feed (Table 2). In the four larvae, ASFV genome copies/mL varied from 10^4.7^ to 10^4.8^. In the collected wash fluids, genome copy number/mL varied from 10^4.7^ to 10^5.9^ (Q1 = 10^4.9^, M = 10^5.1^, Q3 = 10^5.9^, n = 17) (Figure 3A). In the same study, following 24 h exposure to the spiked feed, ASFV DNA was detected in none of the collected and rinsed larvae and in 12% of the wash fluids (Table 2). In the three collected wash fluids, ASFV genome copies/mL varied from 10^4.7^ to 10^4.8^ (Figure 3A). At 1 dpe, viral DNA was detected in 8% of the sampled larvae and 4% of the collected wash fluids (Table 2). Genome copy numbers in these samples were 10^4.7^ and 10^5.2^ (larvae) and 10^4.6^ (wash fluid) copies/mL. No ASFV DNA was detected in larvae and wash fluids collected at 2, 3 and 5 dpe (Table 2, Figure 3A).

In study H2, ASFV DNA was detected in 4% of the rinsed larvae and 12% of the wash fluids collected following both 5 h and 24 h exposure to feed spiked to a titer of ~10^5^ TCID_50_ ASFV/g feed (Table 2). At 5 h, the level of ASFV genome copies/mL in these 4 samples was 10^5.0^ (in the larva) and 10^4.7^, 10^4.8^ and 10^4.9^ (in wash fluids). At 24 h, the level of ASFV DNA was similar with 10^4.7^ (in the larva) and 10^4.8^, 10^4.8^ and 10^5.0^ (in wash fluids) genome copies/mL (Figure 3B). On subsequent sampling days, ASFV DNA was detected in 4% of the larvae sampled at 3 dpe, and in 4% of the wash fluids collected at 1, 2 and 3 dpe. The level of ASFV DNA in these samples was 10^4.9^ (in larva, 1 dpe), 10^4.7^ (in wash fluid, 1 dpe), 10^4.8^ (wash fluid, 2 dpe) and 10^4.9^ (wash fluid, 3 dpe) genome copies/mL. No viral DNA was detected in larvae sampled at 1, 2 and 6 dpe, and in wash fluids sampled at 6 dpe (Table 2, Figure 3B). No attempts were made to isolate infectious ASFV from *H. illucens* larvae, as the Cq values were too high to allow detection of infectious ASFV using our cell culture system [19]. 

In studies H1 and H2, no viral DNA was detected in the control larvae (larvae allowed to consume unspiked feed) or wash fluids obtained from these larvae (data not shown). 

Regarding spiked feed samples collected during study H1, a slight decrease in the amount of viral DNA was observed in spleen suspension kept in the Falcon tube in the environmental chamber at 27 °C from 2 h to 5 dpe. It should be noted, that in spiked feed exposed to the larvae for 24 h, the level of viral DNA was lower (10^5.4^ genome copies/mL), when compared to the level (10^6.2^ genome copies/mL), observed in the spiked feed kept in the Falcon tube for 24 h (Figure 3A). In study H2, a slight decrease in the amount of ASFV DNA was observed in spiked feed samples kept in a Falcon tube in the environmental chamber from 5 h to 6 dpe. The level of viral DNA was lower within the spiked feed exposed to the larvae for 24 h (10^5.4^ genome copies/mL), when compared to the level (10^6.8^ genome copies/mL), detected in the spiked feed kept in the Falcon tube for 24 h (Figure 3B). 

**Figure 4:**
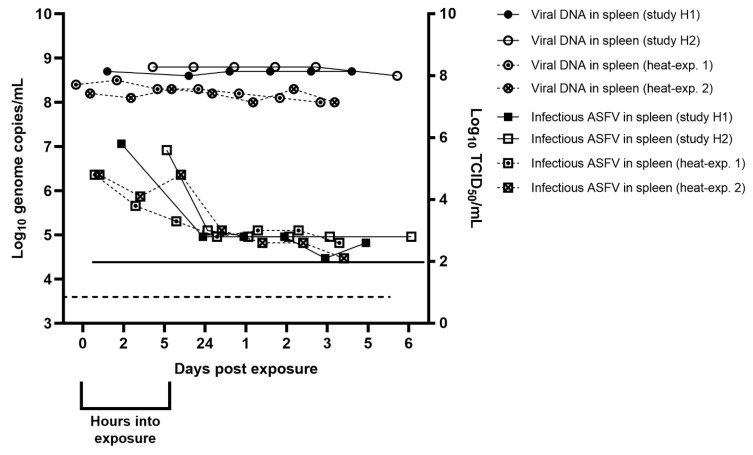
The presence of ASFV DNA and infectious virus in sampled spleen controls. The dashed line indicates the detection limit for the qPCR assay (log_10_ 3.6 copies/mL). The solid line indicates the detection limit for the virus isolation assay (log_10_ 2 TCID_50_/mL).

The level of viral DNA in the spleen suspensions kept in the environmental chamber from 2 h-5 dpe (study H1) and 5 h-6 dpe (study H2) at 27 °C was stable with no apparent decrease throughout the two studies. ASFV genome copies/mL in the samples were between 10^8.6^ to 10^8.7^ (study H1) and 10^8.6^ to 10^8.8^ (study H2) (Figure 4). Quantifying the infectivity of the same clarified spleen suspensions, showed that they had a titer of log_10_ 5.8 TCID_50_/mL at 2 h into exposure (study H1) and log_10_ 5.6 TCID_50_/mL at 5 h into exposure (study H2). The level of infectious virus in the spleen suspensions decreased markedly before the next sampling at 24 h in both studies, with a 3 (study H1) and 2.6 (study H2) log_10_ TCID_50_/mL decrease in titer between samplings. Between 24 h and 5 dpe (study H1) or 6 dpe (study H2), this low level of infectious virus within these samples remained stable (Figure 4). 

The amount of ASFV DNA in the spleen suspension samples kept in the Thermomixer (Eppendorf, Hamburg, Germany) from 0–4 dpe (heat experiments 1 and 2) at 27 °C was stable with no apparent decrease throughout the two experiments. Genome copies/mL in the sampled serum were between 10^8.0^ to 10^8.5^ (heat-exp. 1) and 10^8.0^ to 10^8.3^ (heat-exp. 2) (Figure 4). The spleen suspension samples had a titer of log_10_ 4.8 TCID_50_/mL (heat-exp. 1 and heat-exp. 2) at 0 dpe. In both experiments, the level of infectious virus dropped markedly within the first 24 h of sampling (Figure 4). 

### 3.3. Oral Exposure of Pigs to ASFV-Exposed T. molitor and H. illucens Larvae 

To assess the ability of larvae fed on ASFV to transmit the virus to susceptible hosts, pigs were fed cake containing insect larvae that had been exposed to ASFV. 

In group 1, six pigs were each fed with 50 *T. molitor* larvae that had been euthanized immediately after they had fed on serum containing ASFV. Based on back titration of the serum pool used for insect exposure (see above), it was calculated that each pig in this group received ~10^5.0^ TCID_50_ of ASFV upon oral uptake of the 50 *T. molitor* larvae within soft cake dough. 

Six pigs in group 2 were fed cakes containing 50 *T. molitor* larvae that had been euthanized 48 h after feeding on this serum. In group 3, six pigs were each fed cakes containing 50 *H. illucens* larvae that had been euthanized after 5 h feeding on feed spiked with clarified spleen suspension containing ASFV, and in group 4, six pigs were fed cakes containing 50 *H. illucens* larvae that had been euthanized 24 h after feeding on the same spiked feed. 

Following oral inoculation of the 24 pigs within the four groups, one pig in group 1 (pig 2) died, unexpectedly, at 10 dpe without any prior clinical signs indicative of an ASFV infection (e.g., fever). No ASFV DNA was detected in EDTA-blood obtained from this pig on that day and thus we believe that this death was unrelated to the ASFV-contaminated larvae exposure. None of the remaining 23 pigs in groups 1–4 developed any clinical signs of an ASFV infection. Furthermore, no ASFV DNA was detected in EDTA-blood samples and no anti-ASFV antibodies were detected in sera from these 23 pigs at the end of the study period, at 13 dpe (data not shown). 

## 4. Discussion

We describe here the use of oral virus bioexposure assays for *T. molitor* and *H. illucens* larvae [12] using ASFV. Meat and slaughter byproducts could potentially, in the future, be used as a substrate for insect production as this comes with a huge potential for increasing the circular economy of this production system [1]. Given the current world-wide distribution of ASFV [10], it cannot be ruled out that infectious ASFV could be present within some of these products, as it is known to be a very stable virus, e.g., within meat from infected pigs [6]. The fate of ASFV in insects used for feed is of particular interest. It is the only known DNA arbovirus and can replicate within tick vectors, from genus *Ornithodorus* [20,21]. Furthermore, infectious virus has been detected in insects, i.e., in adult *Stomoxys calcitrans*, for up to 12 and 48 h, respectively, following feeding on blood containing ASFV [22,23]. Additionally, it has been shown that infectious ASFV can be mechanically transmitted to pigs via oral uptake of *S. calcitrans* fed on viremic blood [14]. It cannot be ruled out that the same mode of transmission would be possible when the virus is fed (inadvertently) to insects used for feed—including *T. molitor* and H. *illucens* larvae, that are then subsequently fed to pigs. 

In this study, following feeding on serum from pigs infected with ASFV POL/2015/Podlaskie with a titer of 10^3.3^ TCID_50_/5 μL (equivalent to 10^5.6^ TCID_50_/mL), viral DNA was detected for the entire study period, up to 7 and 9 days, respectively, in exposed *T. molitor* larvae. The length of these studies was based on previous exposure studies with *T. molitor* using a porcine coronavirus in our laboratory. In these earlier studies, two different porcine respiratory coronaviruses (PRCVs) were detected within the *T. molitor* larvae for up to three days post exposure only [12]. As observed for PRCV RNA [12], the amount of ASFV DNA in the sampled larvae was low (Cq values of >30) from the time of exposure, and the number of larvae in which it could be detected decreased subsequently. However, the level of viral DNA within the larvae in which the virus could be detected remained relatively stable, which contrasted with previous findings [12] in which the level of PRCV RNA decreased rapidly and declined to an undetectable level after 3 days. Hence, ASFV DNA seems to be more stable within these larvae than PRCV RNA. 

Following exposure of *H. illucens* to chicken feed spiked with spleen suspension derived from pigs infected with ASFV POL/2015/Podlaskie, with a titer of ~10^5^ TCID_50_ ASFV/g feed, viral DNA was detected for up to 3 days post exposure in the rinsed larvae and their wash fluids. After this, the level of viral DNA within the samples was below the detection limit of the qPCR assay. Cq values were high (>35) in all sampled larvae and wash fluids from the earliest time point, but the levels remained relatively stable within both materials in which the virus could be detected for up to 3 dpe. Using PRCV in the earlier exposure studies, viral RNA was also detectable in *H. illucens* larvae for up to 3 days post virus exposures [12]. It should be noted that for the PRCV studies using *H. illucens* larvae, virus suspension containing serum was used for these virus exposures [12]. As discussed later, this could potentially have an effect on the stability of the viruses used. 

In both *T. molitor* and *H. illucens* larvae, the absence of an increase in ASFV DNA over time suggests that these larvae are not biological vectors for the virus, since there was no indication of virus replication within them. Similar conclusions were made from studies with ASFV using adult *S. calcitrans* [22] and blow fly larvae [24], in which no signs of virus replication were detected within the insects, but they can act as mechanical vectors [14,23]. 

In the *H. illucens* ASFV exposure studies, it was observed that extending the exposure period of the larvae to the spiked feed from 2 h to 5 h or 24 h did not increase the number of larvae in which ASFV DNA could be detected. Indeed, the number of rinsed larvae and wash fluids in which viral DNA was detectable decreased markedly when the exposure periods were extended. Interestingly, Cq values were higher by 3.5 (study H1) and 6 (study H2), respectively, (i.e., lower amounts of DNA were present) in the spiked feed used for the two exposures of the larvae at 24 h (i.e., spiked feed with larvae) when compared to spiked feed kept in the insect incubator for 24 h (i.e., spiked feed without larvae).

It has previously been observed, that *H. illucens* larvae are able to reduce the level of bacteria present in pig manure [25]. The same seems to be true for the level of ASFV genomic material in spiked feed in this study. Previous work has indicated that blow fly larvae can inactivate ASFV within organ material [24]. In that study, viral DNA could be detected in blow fly larvae of the two species, *Lucilla sericata* and *Calliphora vicina*, after feeding on spleen material obtained from ASFV infected pigs, but no infectious virus could be recovered from the spleen after the larvae had fed on it. Infectious virus was, however, recovered from spleen that had not been exposed to the larvae [24]. 

At 5 h into ASFV exposures using spleen supernatant (study H1), the number of rinsed *H. illucens* larvae and wash fluids in which ASFV DNA could be detected was considerably lower than the proportion of RT-PCR positive *H. illucens* larvae that had been exposed to PRCV virus suspensions (containing 10% serum) for 6 and 8 h, respectively, in an earlier study [12]. This could perhaps be attributed to the different materials used for exposures. Based on the results obtained in this study, serum could be hypothesized to have a stabilizing/protective effect on ASFV. In serum and clarified spleen suspensions kept at 27 °C in this study, both in the insect incubator and in the laboratory, the level of infectious ASFV remained stable for a considerably longer time period in serum (48 h and beyond), than in a spleen suspension, in which the level of infectious virus decreased markedly within the first 24 h. A stabilizing effect of serum on ASFV during exposures to different inactivation methods has previously been reported [26]. 

In a previous insect virus exposure study with adult *S. calcitrans* exposed to ASFV with a titer of 10^5.2^ TCID_50_/mL, considerably lower Cq values (below 25.5) were detected in flies sampled immediately after exposure [22] when compared to *T. molitor* larvae and *H. illucens* larvae in this study. The *S. calcitrans* could theoretically have consumed up to 11–15 µL of blood [27]. This means that the flies may have contained 10^3.2^ to 10^3.4^ TCID_50_ ASFV—which compares well with the 10^3.3^ TCID_50_ ASFV/5 µL used for exposure of *T. molitor* larva. As previously discussed [12], the higher Cq values in *T. molitor* (above 30) when compared to *S. calcitrans* (below 25.5) could perhaps be attributed to less efficient extraction of DNA from the larvae prior to qPCR analysis. Even though homogenates of the larvae were diluted 1:5 prior to extraction, the high lipid content of the homogenates could interfere with DNA extraction [12]. 

Attempts to isolate infectious virus from six *T. molitor* larvae was not successful following passaging in PPAM. The six samples had Cq values in the range from 29.8 to 32.8. It has previously been shown that ASFV infectivity assays can be expected to fail (or be unreliable) if Cq values are above 30 [19]. In addition, the six samples were not filtered to remove contaminants. Filtration was attempted, but proved difficult, and for four out of six samples, contamination of the PPAM, e.g., by bacteria and fungi, was observed. It has previously been found that it can be difficult to examine insect samples for infectious virus due to the presence of such contaminators [22]. 

An additional approach to assess the presence of infectious virus within the *T. molitor* and *H. illucens* larvae and the risk of virus transmission via oral consumption was used by feeding pigs with cakes containing larvae exposed to ASFV POL/2015/Podlaskie. Pigs that were allowed to consume 50 *T. molitor* (euthanized immediately after ASFV exposure or at 2 days post virus exposure) or 50 *H. illucens* (euthanized at 5 h or 24 h into ASFV exposure) larvae did not become infected with ASFV. It has previously been shown that the same inoculation method, using oral uptake of blood-fed insects (using pig blood containing ASFV) within soft cake dough, can result in ASFV infection [14]. In the current study, the number of *T. molitor* and *H. illucens* larvae used for feeding of pigs in all four groups was based on the expected dose in the 50 *T. molitor* of 5.0 log_10_ TCID_50_ ASFV used for inoculation of group 1. Based on the number of larvae of both species that contained the virus at these time points and the level of viral DNA within them in studies T1, T2, H1 and H2, it was expected that the dose used for inoculation of the remaining three pig groups was lower. It was not attempted to establish the infectious dose of ASFV in the larvae used for inoculation. In group 1, however, the expected dose used for the six pigs that were exposed to 50 *T. molitor* larvae euthanized immediately after feeding on viremic serum did not differ significantly from the inoculation dose previously used [14]. In that study, two out of four pigs that consumed 20 *S. calcitrans* fed on viremic blood became infected. It was estimated that these 20 flies provided an inoculation dose of 5.1–5.3 log_10_ TCID_50_ ASFV. Oral infection of pigs with ASFV can, however, be difficult to establish under experimental settings, e.g., using feed inoculation [28]. Indeed, in that study, oral inoculation of pigs with feed containing a dose of 4.3–5.0 log_10_ TCID_50_ given on 14 consecutive days did not result in infection. In our study, two weeks following oral inoculation of the pigs, five selected pigs were inoculated intranasally with 10^4^TCID_50_ of the ASFV POL/2015/Podlaskie. These pigs did become infected with the virus 4–5 days following the inoculations, demonstrating that the pigs were indeed susceptible to the infection (data not shown). It has been reported that the minimum infectious dose of ASFV in solid matter (compound feed) is higher when compared to liquid [29]. The reason for this is not known, but it has been suggested that liquids provide a suitable substrate for virus contact with the tonsils where the primary virus replication occurs following exposure via the oronasal or intrapharyngeal routes [29]. However, in a previous study, in which two groups of pigs were fed either intact flies (solid matter) or fly homogenates (liquid form) following feeding of these flies on blood containing ASFV, no difference was observed in the establishment of infection between the two groups of pigs [14]. 

In summary, in this study, we have used established virus bioexposure assays for *T. molitor* and *H. illucens* to assess the survival of ASFV within these larvae. In this study, and the earlier study [12], the detection and survival of viruses within insect larvae seems to depend on many factors, including the virus itself, the material used for exposure and the insects. Therefore, care should be taken with extrapolation of results obtained using one pathogen in one insect species to others. Finally, even though we, in our experimental setting, were not able to demonstrate oral infection of a small number of pigs using *T. molitor* and *H. illucens* larvae that had been exposed to ASFV, it does not fully rule out the possibility of virus transmission via this route in field settings. 

## Figures and Tables

**Table 1:** Number and percentage of *T. molitor* larvae samples in which ASFV DNA was detected on different sampling days in studies T1 and T2.

	Study T1	Study T2
dpe	qPCR Positive	
0	24/25 (96%)	25/25 (100%)
1	9/25 (36%)	11/25 (44%)
2	4/25 (16%)	8/25 (32%)
3	3/25 (12%)	2/25 (8%)
4	6/25 (24%)	1/25 (4%)
6	2/25 (8%)	N.D.
7	N.D.	2/25 (8%)
9	1/25 (4%)	N.D.

The Table shows the number of larvae with ASFV DNA detected/total number tested and the percentage of positive detections (in parenthesis). dpe = days post exposure N.D = no data.

**Table 2:** Number and percentage of *H. illucens* larvae samples and wash fluids in which ASFV DNA was detected on different sampling days in studies H1 and H2.

	Study H1		Study H2	
	Larvae	Wash Fluids	Larvae	Wash Fluids
h/dpe	qPCR Positive		qPCR Positive	
2 h	4/25 (16%)	17/25 (68%)	N.D.	N.D.
5 h	N.D.	N.D.	1/25 (4%)	3/25 (12%)
24 h	0/25 (0%)	3/25 (12%)	1/25 (4%)	3/25 (12%)
1	2/25 (8%)	1/25 (4%)	0/25 (0%)	1/25 (4%)
2	0/25 (0%)	0/25 (0%)	0/25 (0%)	1/25 (4%)
3	0/25 (0%)	0/25 (0%)	1/25 (4%)	1/25 (4%)
5	0/25 (0%)	0/25 (0%)	N.D.	N.D.
6	N.D.	N.D.	0/25 (0%)	0/25 (0%)

The Table shows the ASFV DNA detected/total number and percentage of positive detection (in parenthesis). h = hours into exposure, dpe = days post exposure N.D = no data.

## Data Availability

All necessary data are contained within the article.
